# Bryophytes can recognize their neighbours through volatile organic compounds

**DOI:** 10.1038/s41598-020-64108-y

**Published:** 2020-05-04

**Authors:** Eliška Vicherová, Robert Glinwood, Tomáš Hájek, Petr Šmilauer, Velemir Ninkovic

**Affiliations:** 1 Faculty of Science, University of South Bohemia, Branišovská 1760, CZ-370 05 České Budějovice, Czech Republic; 2 Institute of Botany of the Czech Academy of Sciences, Dukelská 135, CZ-379 82 Třeboň, Czech Republic; 3 Department of Crop Production Ecology, Swedish University of Agricultural Sciences, P.O. Box 7043, SE-75007 Uppsala, Sweden; 4 Department of Ecology, Swedish University of Agricultural Sciences, P.O. Box 7044, SE-75007 Uppsala, Sweden

**Keywords:** Ecology, Plant sciences, Ecology

## Abstract

Communication between vascular plants through volatile organic compounds (VOCs) impacts on ecosystem functioning. However, nothing is known about that between non-vascular plants. To investigate plant–plant VOCs interaction in bryophytes we exposed rare peatland moss *Hamatocaulis vernicosus* to VOCs of its common competitor *Sphagnum flexuosum* in an air-flow system of connected containers under artificial light, supplemented or unsupplemented by far-red (FR) light. When exposed to VOCs of *S. flexuosum*, shoots of *H. vernicosus* elongated and emitted six times higher amounts of a compound chemically related to β-cyclocitral, which is employed in stress signalling and allelopathy in vascular plants. The VOCs emission was affected similarly by FR light addition, possibly simulating competition stress. This is the first evidence of plant–plant VOCs interaction in non-vascular plants, analogous to that in vascular plants. The findings open new possibilities for understanding the language and evolution of communication in land plants.

## Introduction

Interactions are crucial for the survival of individuals in ecological communities^1^. Consequently, animals and plants perceive a variety of cues by which they can ascertain what is in the proximity. Until the end of the twentieth century, however, the active sharing of information seemed solely the domain of animals. Plants were viewed as passive, stationary organisms, with only basic interactions with other organisms^2^, apart from pollinators. With the discovery of plant communication^3,4^, it became evident that plants use light^5^, touch^6–9^, vibrations^10^ and chemicals^11–13^ to communicate in an intricate web of multitrophic interactions that affect functioning of ecosystems.

Volatile organic compounds (VOCs) are involved in communication in eukaryotic and prokaryotic organisms including animals and vascular plants^14^, bacteria^15^, brown algae^16^, and fungi^17^. These secondary metabolites with low molecular weight and high vapour pressure at ambient temperature can move freely through the air. They are produced in cytosol (organelles or cytoplasm) and are possibly transported outside the cell through lipophilic carriers (in aqueous environments of cytosol and cell wall) and ABC transporters (through lipophilic plasma membrane^18,19^). The production of VOCs by plants depends on genetic identity of the individual, life history and health, plant organ, photoperiod, light quality (e.g., red to far-red (R/FR) ratio), symbiotic organisms and other factors^1,20–23^. Hence, each organism has a specific VOC blend including compounds unique for the given taxon^24^ as well as chemicals with specific ecological meaning (e.g.^25^). Species that can detect and decipher the encoded information can use VOCs in interactions, as a source of information.

Plant–plant VOC interaction often takes the form of eavesdropping. Plants can estimate the strength of their neighbouring competitors and, accordingly, adjust their growth^26^. Parasitic plants can use VOCs to locate their hosts^24^. VOCs could even be used as indicators of unfavourable environmental conditions^15,27^ that eavesdroppers survive better by inducing tolerance or resistance to the stress. Yet VOC production in plant–plant interactions may be beneficial for the emitter itself, e.g., when it serves as a quick information transfer between different plant parts, particularly in plants that are unable to transmit that information through vascular tissue (e.g. desert and semi-desert plants^28^). Similarly, VOCs can be used as cues of impending danger, where the danger is averted more easily when plants employ inter- or intraspecific interactions (e.g. reducing plant attractiveness for herbivores and limiting their population development^29^, and by attracting predators of herbivores^30^).

Our knowledge about plant communication has been gathered almost solely from angiosperms, particularly crop species^14^, and information about other plant groups is limited or lacking. We know that gymnosperms can communicate through volatiles^31^, however, we know nothing about phylogenetically more basal groups of vascular plants (such as ferns) and nonvascular plants (green algae, bryophytes).

To our knowledge, plant–plant volatile interactions has never been studied in bryophytes. There are indications that mosses might use VOCs in interactions in similar ways as vascular plants do; in animal-mediated pollination and seed dispersal, mosses can use odours to facilitate spore and spermatozoid dispersal. Some of the coprophilous mosses (family *Splachnaceae*) are entomophilous, i.e. they use brightly coloured, scented sporophytes to attract flies that disperse their spores to suitable substrate^32^. Similarly, fertile female shoots of at least some moss genera produce odours more attractive to microarthropods than the rest of the population, facilitating spermatozoid dispersal^33^.

The basic interaction with insects and microarthropods suggests bryophytes might be able to communicate through VOCs on a sophisticated level. Hence, we hypothesize that, similarly to angiosperms, bryophytes can use VOCs to evaluate the competitive strength of their neighbours and adjust accordingly their shoot growth to avoid competitive exclusion. Competition among bryophytes for light and other resources is tightly linked with their poikilohydry. To maintain hydration, bryophytes often grow in a dense layer (cushions, mats) where light penetrates only one or two centimetres below the surface and the competition is manifested more like a *competition for space*^34^. If an individual grows more slowly than its neighbours, it becomes shaded into darkness; when it overgrows its neighbours, it becomes limited by desiccation. Similar to vascular plants, bryophytes detect spectral changes of light after passing through vegetation^35^ that absorbs photosynthetically active light but transmits FR light. However, this mechanism alone cannot distinguish between shading by vascular plants or by overgrowing shoots of a competitor in the bryophyte layer. Thus, individuals with the ability to recognize the identity of the overshadowing neighbour could have an evolutionary advantage.

If our hypothesis is valid, we may conclude that the capacity to use volatile cues as information in neighbour detection, as we know it from angiosperms, may be, in at least some form, shared by all land plants. We used a pair of competitor moss species from fens, bryophyte-dominated minerotrophic peatlands, to test the following hypotheses:

- *Hamatocaulis vernicosus* (Mitt.) Hedenäs (a rare moss species protected by European law, Natura 2000) will increase its growth in length when exposed to VOCs from its natural competitor *Sphagnum flexuosum* Dozy & Molk. to avoid being out-competed.
- Volatiles released by S. *flexuosum* will change the VOC production of *H. vernicosus*, possibly as a cue for surrounding *H. vernicosus* individuals. Such a response has been observed in vascular plants^23^.
- Light quality (increased proportion of far-red light imitating shade by vegetation) will affect VOC production in both species and increase their growth in length, as seen in vascular plants^22^.

## Results

### Sphagnum flexuosum volatiles affect growth of Hamatocaulis vernicosus

*H. vernicosus* changed its growth pattern when exposed to VOCs produced by *S. flexuosum*. While the overall biomass production remained unchanged (Fig. S3), the shoots increased growth in length but only when the light treatments were pooled together (F_1,5_ = 8.8, p = 0.031), about 0.3 cm and 0.5–0.7 cm in 30 days under normal and supplemented far-red light (FR− and FR+; Fig. 2). The increased growth in length was not significantly compensated by lower shoot branching under FR− (F_1,2_ = 1.51, p = 0.34, Fig. S4) or FR+ (F_1,3_ = 0.06, p = 0.82, Fig. S4). In contrast, FR light induced creation of short branches (F_1,7_ = 7.2, p = 0.031, Fig. S5). Surprisingly, the FR+ did not induce greater growth in length of *H. vernicosus* shoots (F_2,5_ = 2.6, p = 0.17, Fig. S6); however, it induced higher growth in length of *S. flexuosum* shoots (F_1,4_ = 21.0, p = 0.01; Fig. 3) without changing overall biomass production (F_1,4_ = 4.0, p = 0.12, Fig. S7).

**Figure 1:**
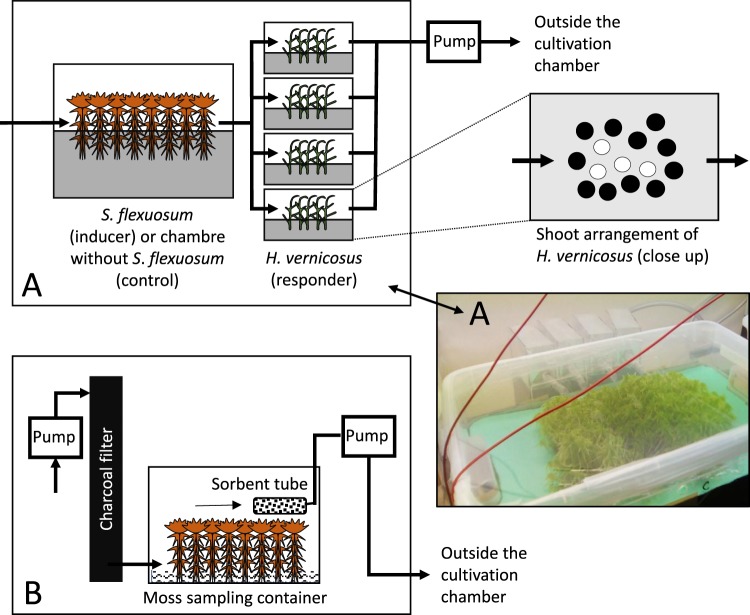
Experimental setup of (**A**) One of the *Cultivation units* inside the cultivation chamber (Fig. S1) and (**B**) VOCs sampling design. (**A**) Cultivation unit: The air was drawn through the inducer (*Sphagnum flexuosum*) or control (without *S. flexuosum* chamber to the four responder (*Hamatocaulis vernicosus*) chambers and pumped out of the cultivation chamber. *H. vernicosus* grew on floating mat bearing 15 holes, each accommodating three shoots of *H. vernicosus*. The four white circles in *H. vernicosus* plate indicate shoot triplets used for growth measurements. (**B**) VOCs sampling: The air was pumped through a charcoal filter over the moss carpet. Air enriched by VOCs was drawn through an adsorbent (Porapak tube) and then vented outside the cultivation chamber.

**Figure 2:**
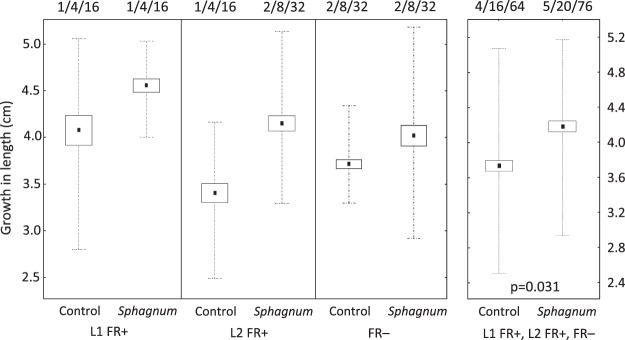
The length increment of *H. vernicosus* shoots grown under artificial light without FR light addition (FR−) and added FR light (L1 FR+, L2 FR+) in cultivation units (Fig. 1) for 30 days (L2 FR+ had more blue light than L1 FR+, see methods for details). The shoots were exposed to VOCs produced by surrounding *H. vernicosus* individuals and to VOCs from *S. flexuosum* chamber (*Sphagnum*) or chamber without *S. flexuosum* (*Control*). The box and whiskers depict ± s.e. and minimum/maximum values, the numbers above depict number of inducer chamber/responder chamber/*H. vernicosus* replicates. The *H. vernicosu*s growth increment increased significantly when the shoots were exposed to *S. flexuosum* VOCs (F_1,5_ = 8.8, p = 0.031, tested across all light treatments; the experimental design and number of replicates did not allow to test the light treatments individually).

**Figure 3:**
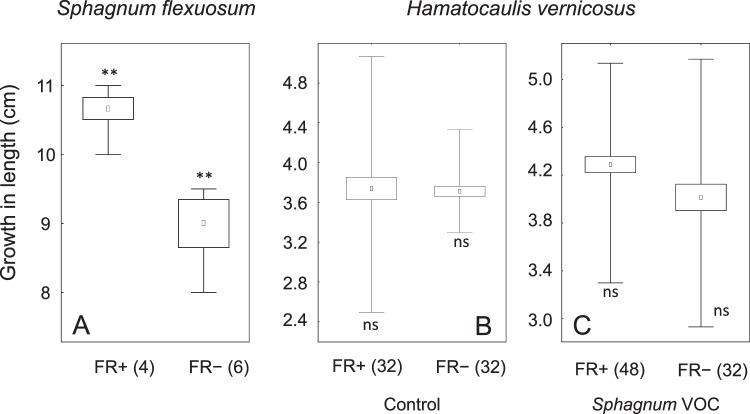
Growth in length of *S. flexuosum* (**A**) and *Hamatocaulis vernicosus* (**B** – control shoots, **C** – shoots exposed to *Sphagnum* VOCs) cultivated in growing chamber in cultivation units (Fig. 1) under artificial light without FR light addition (FR−) and added FR light (FR+) for 30 days. The box and whiskers depict ± s.e. and minimum/maximum values, the numbers beside light treatments depict number of replicates. Significant differences between treatments (**P = 0.01; ANOVA test).

### S. *flexuosum* volatiles affect *H. vernicosus* VOCs emission

**Figure 4:**
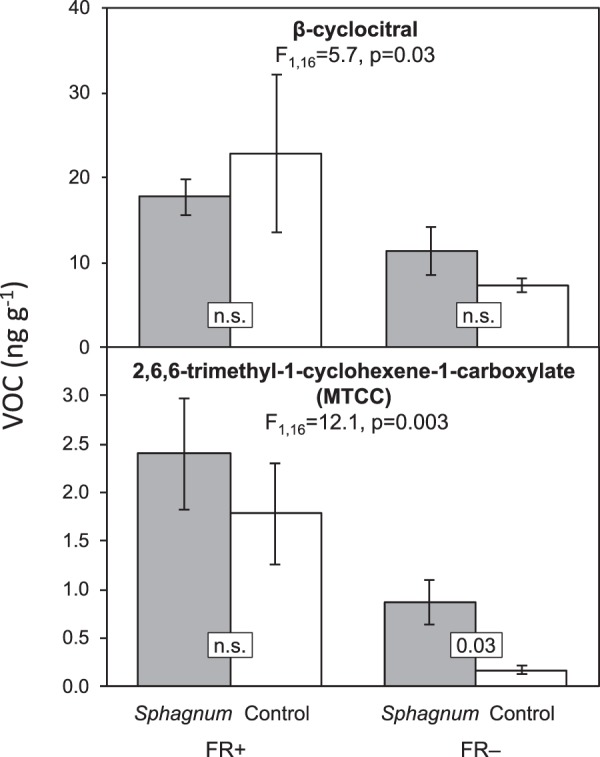
Quantity of volatile organic compounds (ng g^−1^) whose production by *Hamatocaulis vernicosus* carpets was influenced by FR light addition (MTCC, B-cyclocitral) or exposure to *Sphagnum* VOC (MTCC under FR−). *H. vernicosus* shoots in the carpets were exposed only to VOCs released from neighbouring *H. vernicosus* individuals (control) or to VOCs released by surrounding *H. vernicosus* individuals and to VOC blend from *S. flexuosum* carpet (*Sphagnum* exposure), for more details see methods, Fig. S1 and Table S2. Both species were cultivated under artificial light conditions without FR light addition (FR−) or added FR light (FR+). VOCs were collected for 72 h. The error bars depict ± s.e. of means.

In addition to growth changes, VOCs emitted by *S. flexuosum* induced changes in VOCs composition of *H. vernicosus*. Specifically, *S. flexuosum* VOCs induced six times higher emission of methyl 2,6,6-trimethyl-1-cyclohexene-1-carboxylate (MTCC) under FR− (F_1,4_ = 10.3, p = 0.032, Fig. 4), the production of the other 23 detected compounds remained unchanged (Table S2). The changes were not observed under FR+, probably because the FR light itself increased this compound 12 times (Fig. 4, control). The total amount of VOCs released by *H. vernicosus* was not affected by VOCs from *S. flexuosum*.

### FR light changes *H. vernicosus* and *S. flexuosum* VOCs emission

FR light did not induce production of new volatile compounds nor change the total amount of VOCs produced. However, it significantly increased production of specific VOCs in both species. *S. flexuosum* emitted higher amounts of nine VOCs (β-cyclocitral, F_1,10_ = 95.6, p < 0.0001; MTCC, F_1,8_ = 67.8, p < 0.0001; unknown compounds 29, 30, 23, 31, 33 p = 0.02–0.004) when grown continuously under FR light (Tables 1, S3). Emission of most of these compounds remained high even after the FR light had been switched off (Table 1). In addition, switching off the FR light increased emission of two other compounds, unknown compounds 27 and 35. *H. vernicosus* reacted to FR+ by an increased production of β-cyclocitral and MTCC (F_1,16_ = 5.7, p = 0.03 and F_1,16_ = 12.1, p = 0.003 respectively, Fig. 4), compounds that had increased emission in *S. flexuosum* under the same conditions.

**Table 1:** Quantity of significantly FR light-dependent volatile organic compounds (ng g^−1^) produced by *Sphagnum flexuosum* cultivated under artificial light conditions without FR light addition (FR−) and supplemented FR light (FR+). The volatiles were sampled under artificial light conditions (Standard sampling) or FR light was added to shoots exposed to FR light during cultivation experiment (FR light sampling). (One-way ANOVA performed separately for each compound and sampling treatment, *p < 0.01, **p < 0.05.) VOCs were collected for 72 h.

*Sphagnum flexuosum*	standard sampling		FR light sampling	
	FR+	FR−	FR+	FR−
methyl 2,6,6-trimethyl-1-cyclohexene-1-carboxylate	2.85 (±0.25)	0.27 (±0.70)**	2.69 (±0.65)	0.31 (±0.08)**
β-cyclocitral	5.55 (±0.40)	0.98 (±0.38)**	4.52 (±0.90)	0.80 (±0.18)**
unknown 23	2.65 (±0.48)	0.90 (±0.52)*	15.40 (±4.96)	2.96 (±0.99)*
unknown 27 (possible sesquiterpene)			0.91 (±0.12)	0.11 (±0.03)**
unknown 28 (possible sesquiterpene)	0.47 (±0.06)	0.08 (±0.02)**	1.58 (±0.38)	0.18 (±0.08)**
unknown 29 (possible sesquiterpene)	1.07 (±0.15)	0.19 (±0.06)**	2.73 (±0.70)	0.17 (±0.04)**
unknown 30	1.68 (±0.21)	0.33 (±0.12)**	5.45 (±1.34)	0.52 (±0.14)**
unknown 31	8.17 (±1.48)	2.97 (±1.07)**	4.81 (±0.83)	1.72 (±0.46)**
unknown 33			5.45 (±1.40)	0.51 (±0.14)**
unknown 35	3.89 (±0.44)	2.58 (±0.83)*		

### VOCs blend of S. flexuosum and H. vernicosus

In total, we detected 29 VOCs produced by *S. flexuosum* and 24 VOCs produced by *H. vernicosus* (Tables S2, S3, S4). Four compounds (β-cyclocitral, MTCC, α-copaene and unknown compound 4 (likely a sesquiterpene) were emitted by both species. Except for MTCC, which was produced in similar quantities by both species, the production of individual volatiles was 6–10 times higher in *H. vernicosus* than in *S. flexuosum*. Similarly, the total VOCs production of *H. vernicosus* was approximately four times higher than that of *S. flexuosum*.

## Discussion

The results show that a non-vascular plant, the moss species *Hamatocaulis vernicosus* can detect VOCs from their neighbour. These volatile cues could potentially be used to evaluate the competitive strength of the neighbour. The air-borne volatiles may serve as growth rate cues for nearby bryophyte eavesdroppers that use the information in regulating their own growth. This type of plant–plant interaction observed in bryophytes resembles responses discovered in vascular plants^22,26^ and suggests that plant–plant volatile interaction is developed in the whole Embryophyta division.

### *H. vernicosus* changes growth and VOCs emission in response to volatiles produced by *S. flexuosum*

The accelerated growth in length of *H. vernicosus* in response to *S. flexuosum* VOCs closely resembled a shade-avoidance syndrome that plants, including bryophytes, use as a survival strategy against overshadowing neighbours^36^. The physiological mechanism of shade avoidance has been traditionally connected with a plant’s ability to perceive changes in spectra and intensity of the radiation reflected by foliage of adjacent plants through photoreceptors (i.e. low R/FR ratio, lower amount of blue light). We have demonstrated that shade avoidance is also connected with VOCs detection, at least in bryophytes, where the survival of individual shoots is strictly dependent on keeping the growing apex in the upper illuminated part of the bryophyte canopy. While vascular plants react to VOCs from neighbouring competitors with changes in growth strategy^22,26^, increased growth in length has not been reported; thus, the role of VOCs perception in the shade avoidance syndrome of vascular plants is unclear.

Apart from growth changes, *H. vernicosus* reacted to *S. flexuosum* VOCs by altering its own VOCs emission, specifically increasing production of a compound tentatively identified as methyl 2,6,6-trimethyl-1-cyclohexene-1-carboxylate (MTCC). We tentatively identified MTCC based on matching in a commercial mass spectral library (NIST 2008), since no authentic standard was available. The tentative compound does however appear to share structural similarity with β-cyclocitral (2,6,6-trimethyl-1-cyclohexene-1-carbaldehyde), which was also released by *H. vernicosus* (and confirmed with an authentic standard). To our knowledge, MTCC has not been previously reported as a plant-produced volatile compound, however emission of β-cyclocitral by a moss, and compounds with structural similarity to MTCC have been reported^37,38^. Roles for β-cyclocitral in plant stress signalling^39^ and allelopathy^40^ have been described, and it is conceivable that the structurally related MTCC has similar activity.

The alteration of VOCs blend in response to volatiles from herbivore or pathogen-damaged^41–43^ and undamaged neighbours^29,44,45^ has been documented in vascular plants. The change can be beneficial for both the emitters and receivers upon engaging in tritrophic interactions. For example, volatiles received from emitters via eavesdropping evoked changes in terpenoid^29,44,45^ or alkane^29^ production by receivers, making their VOCs blend less attractive for herbivores (or pathogens) and more attractive for herbivore predators, thus protecting the whole plant community. Since bryophytes are known to have a large variety of terpenoid secondary metabolites with repellent (or even toxic) effects on herbivores and pathogens^38^, their involvement in VOCs interaction is plausible.

Similar principles of cooperation and warning might be expected in bryophyte communities when dealing with competition. Peatland bryophytes (including *H. vernicosus*) have a clonal growth strategy creating genetically identical clusters in the moss layer^46^. Since bryophytes compete predominantly for space^47,48^, species forming mats can withstand competition from a stronger competitor for longer than individual shoots. Consequently, the use of VOCs as stress warning cues between conspecific or even genetically identical neighbours would enhance survival of the micropopulation. As the cue is passed among closely related individuals, transfer of the information is much more efficient than if it would be carried to distant relatives or to different species^21^.

### Volatile organic compounds responsible for bryophyte interaction

Although plant communication has been studied for more than 30 years, the principles behind a ‘language’ of plant signalling remain unclear, particularly regarding competitive interactions. Our study, as well as previous studies^22,26^ clearly shows that plants adjust their growth in response to VOCs from neighbouring plants. However, it is still unknown in what situations VOCs carry information about an emitter’s genetic identity and to what extent a receiver (other than parasitic plants^24^) can evaluate the information. Alternatively, the VOC blend might represent some general cue about a neighbour’s presence or other traits characterizing an emitter’s competitive strength.

The identity of chemical compounds (or blends) responsible for information transfer in volatile interaction is also speculative. Runyon (2006)^24^ showed that, at least in some situations, the bearers of taxon-specific information in plant–plant signalling are terpenoids. In our study we isolated 29 volatiles produced by *S. flexuosum* that mostly differed from those emitted by *H. vernicosus*. The compounds we were able to tentatively identify were terpene-related. Apart from (+)-cyclosativene they are known to be produced by other mosses or liverworts^49–53^. Consequently, if the VOCs blend carried information about the genetic identity of *S. flexuosum* and the information was encoded by VOCs detected in our study, the key part of the cue could be (+)-cyclosativene, one of the unidentified compounds or a specific combination and/or concentration of the detected chemicals. A large number of terpenoid compounds have been identified from bryophytes, but relatively few from the mosses^38^, and little mass spectral data are reported. Further, volatile emission by *Sphagnum* species has not been studied in detail, limiting our ability to identify VOCs specific for the genus *Sphagnum* or even *S. flexuosum*.

A chemical compound considered as a potential cue to indicate future plant competition but not analysed in our study is the plant hormone ethylene. Ethylene, in concentrations physiologically active in vascular plants: (i) had no effect on growth of a moss *Fontinalis squamosa*^54^, (ii) reduced growth of a moss *Physcomitrella patens*^55^ and (iii) inhibited auxin-evoked seta elongation in a liverwort *Pellia epiphylla*^56^. Therefore, ethylene seems to have negative effect on shoot elongation in bryophytes and is unlikely to be responsible for the observed elongation of *H. vernicosus*. The airflow and the low amount of emitter biomass (less than 35 g of DM) in our study may have prevented the build-up of ethylene to physiologically active concentrations 1 ppb^57^ or the even higher concentrations reported to affect experimental plants in previous studies^22,57–59^.

### Bryophytes change VOCs emission in response to light quality

Our mosses did not reduce their total VOCs production when grown under light with a low R/FR ratio, i.e. illumination simulating shading by vegetation. This contrasts with the response of vascular plants to low R/FR ratios^22,60^. However, similar to vascular plants, both mosses changed the composition of their VOCs blends, increasing production of β-cyclocitral and MTCC as well as several unidentified compounds. MTCC concentration was also increased on receiving volatiles of a stronger competitor and may potentially function as a common volatile cue of competition in bryophytes.

Low R/FR light generally evokes shade avoidance syndrome in bryophytes^61,62^ and, accordingly, it led to a strong shoot elongation in *S. flexuosum* in our study. In contrast, the growth of *H. vernicosus* was not significantly affected by supplemental FR light. A lack of response to FR light has been previously recorded in bryophytes^35^. Moreover, it is known that different populations of the same taxa can react differently to low R/FR^63^. Thus, it is possible to conclude that the strength of the response to shading differs between species or even populations and might be influenced by light conditions in the current microhabitat^63^.

The response of bryophytes to R/FR ratio in our experimental system was affected by blue light, emitted in different quantity from the two types of fluorescent tubes. Besides R/FR-sensitive phytochromes, plants detect canopy shade as blue light attenuation via blue light-sensitive cryptochromes. Although each type of photoreceptor has its own signalling pathway, the final response is a result of their integration^64,65^. Consequently, elevated blue light inhibits elongation evoked by low R/FR ratio in vascular plants^59,66,67^. This explains why both control and *Sphagnum*-exposed *H. vernicosus* shoots had slightly lower (though not significantly) elongation rates under light sources richer in blue light (L2FR+).

### Prospective model of plant–plant interactions by VOCs in bryophyte communities

The *Sphagnum*–*Hamatocaulis* interaction reported here demonstrates that bryophytes can use VOCs as warning cues in detection of future competition. This may be one of the cues enabling centuries-long species coexistence in stable bryophyte communities such as in peatlands^68,69^. There, competitive exclusion is thought to be avoided by short-term, often seasonal fluctuations in ecological factors (e.g., water availability and chemistry) that alternately favour individual species^48,70^. Consequently, the ability to detect and interpret VOCs emitted by a stronger competitor may provide the weaker ‘eavesdropper’ with an ecological advantage, enabling it to match its growth with the stronger neighbour and thus bridge the short time span of unfavourable conditions.

Although there are similarities between plant–plant VOCs interactions in bryophytes and vascular plants, these two fundamental groups of land plants differ principally in their ecological strategies. Lacking well-developed anatomical structures allowing efficient water management (vascular tissues, stomata, cuticle), bryophytes must rely on biochemical adaptations to cope with desiccation and related environmental stresses. Therefore, we postulate that VOCs emitted by desiccated or repeatedly rehydrated bryophyte shoots might be decoded as warning cues providing the receivers with time for biochemical acclimation (hardening), since bryophyte desiccation tolerance is largely an inducible trait^71^. Analogous responses to VOCs emitted upon environmental stress are known in vascular plants^23,27^. Consequently, the ability to eavesdrop on desiccation-mediated VOCs cues would present clear ecological advantages, preventing diebacks during drought^72^.

## Conclusions

Our results provide the first evidence of VOCs-mediated interspecific plant–plant interaction in bryophytes, a phylogenetically basal group of land plants. Since the interaction closely resembles that in vascular plants (morphological response to VOCs stress cues, changed VOC blend of the responder, similar chemistry of VOCs cues), one might speculate it evolved in a common ancestor of land plants. Future research on VOC-mediated interactions among bryophytes dealing with biotic (competition, pathogenesis) and abiotic (water) stress may shed new light on the functioning of bryophyte communities and bryophyte-dominated ecosystems.

## Materials and Methods

### Moss material

Bryophyte plant–plant VOCs interactions were studied in a laboratory experiment using artificial poor fen solutions and an air-flow system. We selected two fen moss species – *Hamatocaulis vernicosus* (Mitt.) Hedenäs (rare, Natura 2000 protected species with an optimum in rich fens) and *Sphagnum flexuosum* Dozy & Molk. (strong competitor dominating poor fens). The species naturally coexist in (moderately) rich fens (terminology follows)^73^; *H. vernicosus* grows in hollows and low hummocks, *S. flexuosum* occupies low and high hummocks. If the pH and [Ca^2+^] are lowered in the moss carpet, *S. flexuosum* can outcompete *H. vernicosus* and slowly switch the moderately rich fens to poor fens^74,75^. *H. vernicosus* was used as responder, *S. flexuosum* as inducer. Each species was collected from two fens in South or West Bohemia, Czech Republic (detailed description in Table S1; *H. vernicosus* is locally common in sampled localities and the *H. vernicosus* collection did not endanger local populations).

### Cultivation experiment

*H. vernicosus* and *S. flexuosum* were cultivated in an air-flow system of connected transparent containers placed in a growth chamber. Containers for inducers (*S. flexuosum*/empty plate = control) were made from 22-L polyethylene boxes (36.5 × 25.5 × 26.5 cm, Ikea), and containers for responders (*H. vernicosus*) from 600 mL polypropylene bottles with cut upper parts (11.5 × 3.5 × 15.0 cm, Tissue Culture Flask, Sarstedt). The containers were sealed by transparent polyethylene film secured by paraffin film. Adhesive properties of the film together with slight negative pressure in the container (created by air flow) prevented unwanted air escape to the growth chamber. Each inducer container was connected by transparent polyethylene tubes with four responder containers, creating an individual *container unit* (Fig. 1).

Containers were filled with artificial poor-fen solution (K – 0.8 mg L^−1^, Ca – 0,8 mg L^−1^, Mg – 0,5 mg L^−1^, N – 1.4 mg L^−1^, P – 0,5 mg L^−1^, Cl – 1,4 mg L^−1^, Mn – 5.4 µg L^−1^, B – 5.3 µg L^−1^, S – 1.4 mg L^−1^, Na – 1 mg L^−1^, I – 1 µg L^−1^, Zn – 1 µg L^−1^, Br – 0.9 µg L^−1^, Co – 0.8 µg L^−1^, Cu – 0.7 µg L^−1^), replaced every 9 days. Each responder/inducer container contained 400 mL/17 L of the solution. Lower walls of the containers were darkened to suppress algal growth. Shoots of *H. vernicosus* and *S. flexuosum* were arranged in their natural density into holes made in thin plates of expanded polystyrene floating above the solution. The arrangement ensured sufficient water supply to shoot’s apical parts so moisture would not be growth-limiting. *H. vernicosus* carpet had an oval shape and was composed of 45 apical shoot fragments (16 mm long) growing in 15 holes 0.5 cm apart (three fragments per each hole, Fig. 1). The bed of *S. flexuosum* was rectangular (20 × 15 cm) and composed of approximately 20 mm long apical fragments (about 10 mg on dry mass basis; one or two shoots per hole, holes 0.8 cm apart).

Air flow was created by a pump producing unidirectional flow of approximately 0.1 L min^−1^. The air inlet of inducer containers was at the level of moss shoots. Air was drawn through the bed of *S. flexuosum* (or a control chamber with solution and empty plate) and via connecting tubes to the responder chamber through the *H. vernicosus* stand. The air from the responder chambers was then vented from the room. Consequently, *H. vernicosus* individuals were exposed to VOCs emitted by shoots of either surrounding *H. vernicosus* (inducer chamber without *S. flexuosum*) or to both, inducer and surrounding *H. vernicosus*.

The growth chamber was illuminated by fluorescent lamps with 14:10 h light:dark. Temperature in the room was 23 ± 1 °C and 25 ± 1 °C in the containers around the mosses. The intensity of photosynthetically active radiation at the moss cover was approximately 120 µmol m^–2^ s^–1^. In addition to artificial day light, some of the container units (FR+ treatment) were supplemented by far-red (FR) light of 730 nm (one 10-W SMD LED module per container unit) that resulted in R/FR ratio of 0.23.

The growth chamber was equipped with two models of fluorescent tubes of slightly different light spectra: Osram L 36 W/865 Lumilux Cool Daylight (colour temperature 6500 K) and Osram FQ 80 W/840 HO Constant Lumilux Cool White (4000 K), Germany; the light colour 865 having about two times higher blue light emission than 840 (Figs. S1, S2). Using tubes of different colour temperatures was originally not intended but the experimental design required entire capacity of the growth chamber where the two types of illumination were constructed independently. However, this arrangement allowed us to test the side effect of blue light on plant elongation and volatiles emission. Tubes of both colours provide light of high R/FR ratio. Although fluorescent tubes emit light of partly discrete spectral lines, tubes of both colours have been successfully used in small-scale cultivation for decades (now being replaced by LED-based light sources with continuous light spectra). The placement of the lamps and container units in the chamber was designed to minimise spatial differences in light quality.

Four container units (two with inducer, two controls) were placed in artificial daylight (FR−; *S. flexuosum* unit and control unit under each lamp type), two *container units* (one with *S. flexuosum*, one control) were placed under Osram FQ 80 W/840 with added FR light (L1FR+ treatment) and three container units (two with *S. flexuosum*, one control) under Osram L 36 W/865 with added FR light (L2FR+ treatment; Fig. S1). Each container unit encompassed 16 *H. vernicosus* triplets (i.e. replicates) used for statistical analysis of fragments growth in length, biomass production and branching, and a *S. flexuosum* carpet (divided to two parts, i.e. 2 replicates) used for statistical analysis of fragments growth in length and biomass production. Bryophytes were cultivated under the described conditions for 30 days, except for period of *H. vernicosus* VOCs collection (21–23 and 28–30 day of cultivation).

### VOCs collection

VOCs emitted from *H. vernicosus* and *S. flexuosum* were sampled by dynamic headspace collection (air entrainment). Prior to the entrainment, sampling containers were cleaned with detergent (TEEPOL, 1% w/w) and rinsed with acetone and distilled water. Glass tubes (5 mm diameter) containing the adsorbent Porapak Q (50 mg, mesh 50/80, Supelco, Bellefonte, PA, USA) were cleaned with redistilled dichloromethane and baked overnight at 140 °C under nitrogen flow. Charcoal filters (SGE Analytical Science, Victoria, Australia) were baked overnight at 180 °C under nitrogen flow. PET (polyethylene terephthalate) oven bags (Toppits, Klippan, Sweden) were baked for 2 hours at 140 °C, sampling containers and Teflon connecting tubes were baked overnight at 180 °C.

VOCs sampling was conducted under controlled environment conditions (21 °C, 14/10 h of artificial light/dark). Sampling containers for *S. flexuosum* were made from modified 450 mL glass beakers, sealed by a Petri-dish. Each container contained half a *S. flexuosum* carpet from a container unit (described above). Sampling containers for *H. vernicosus* were made from Duran laboratory glass bottles sealed with material cut from the PET oven bags, each containing all four *H. vernicosus* plates from a container unit (described above). To avoid desiccation, a small volume of nutrient solution was added to the mosses each day during the VOCs collection.

Charcoal-filtered air was pumped into each container at 400 mL min^−1^ and VOCs-enriched air was drawn out through the Porapak tubes at 300 mL min^−1^ (Fig. 1). The difference in flow rates created a slight positive pressure, minimizing entry of unfiltered air. Volatiles were collected over a period of 72 h. Volatiles from *H. vernicosus* were collected on days 21–23 and 28–30 of cultivation, and volatiles from *S. flexuosum* were collected 1–3 and 5–7 days after the end of the cultivation. *S. flexuosum* carpets remained in the collecting chambers between samplings. *S. flexuosum* shoots from the FR light treatment were exposed to FR light during and 24 h prior to the second VOC collection, while the first collection was conducted without FR light supplement.

### VOCs analysis

VOCs were eluted from Porapak tubes with 750 µL redistilled dichloromethane. An internal standard (1-nonene at 20 ng µL^−1^ in the sample) was added and the sample was concentrated to 50 µL under nitrogen flow.

Compounds were identified using coupled gas chromatography/mass spectrometry (GC/MS) as previously described^76^. A 1 μL aliquot of each sample was injected onto a HP-1 column (30 m, 0.25 mm i.d., and 0.25 μm film thickness; J&W Scientific, Santa Clara, CA, USA) housed in a 7890 A gas chromatograph (Agilent Technologies, Santa Clara, CA, USA) coupled to an Agilent 5975 C mass spectrometer. Ionization was by electron impact at 70 eV. The oven temperature was held at 30 °C for 1 min, then programmed at 5 °C min^−1^ to 150 °C, then at 10 °C min^−1^ to 250 °C. The carrier gas was helium with a flow rate of 1 mL min^−1^. Identifications were made by comparison of spectra with a commercial database (NIST 2008) and by comparing mass spectra and retention times with those of authentic standards where available. Most compounds emitted by both species did not generate a satisfactory match in the commercial database making identification unfeasible; these are designated as ‘unknown compound’. Some compounds generated strong matches in the database but authentic standards were not available; these are designated as speculative identifications. Full mass spectral data along with retention indices (Kovats) for all compounds quantified are provided in Supplementary Tables S3, S4.

Compounds were quantified using gas chromatography (GC). A 1-μL aliquot of each sample was injected onto a HP-1 column (dimensions as for GC/MS) housed in a 6890 GC (Agilent Technologies). The temperature program was as for GC/MS and the carrier gas was hydrogen. Compounds were quantified using the internal standard. The entrained moss material was oven dried (60 °C, 24 h) after the final VOCs collection and VOCs amounts were expressed in relation to moss dry mass (ng g^−1^).

### Moss growth measurement

The effect of FR light and *S. flexuosum* volatiles on the growth of *H. vernicosus* was evaluated as weight and length increments and number of new branches in four triplets of *H. vernicosus* fragments that grew in the middle of *H. vernicosus* floating mat (Fig. 1). The fresh mass (FM) was weighed after careful blotting the fragments between sheets of cellulose filter paper and was transformed to dry mass (DM) by the formula: FM = 3.38 × DM following^75^. The growth response of *S. flexuosum* to FR light was evaluated as shoot length increment.

### Statistical analysis

The effect of VOCs and FR light on growth and branching of *H. vernicosus* and the effect of FR light on *S. flexuosum* growth in length and biomass production was evaluated by linear mixed-effect models (LMM, package nlme^77^, in the R statistical language (version 3.4.0; 2017-04-21). Experimental design of *container units* was reflected in the model specification (responder’s container nested in inducer’s container, both factors were used as random factors). Since the growth of *H. vernicosus* was not affected by light treatments, the effect of *S. flexuosum* VOCs on growth of *H. vernicosus* (length, weight) was evaluated across the two light treatments (FR+, FR−), reducing the problem with a low number of replicates induced by the design complexity.

The effect of *S. flexuosum/H. vernicosus* VOCs production and the effect of *S. flexuosum* VOCs on *H. vernicosus* VOCs production was evaluated by one-way analysis of variance (ANOVA) in a program Statistica (ver. 8). The evaluation of VOCs production was done individually for each VOCs compound. The two *H. vernicosus* VOCs samplings were pooled together, as well as the two FR treatments of different artificial daylight quality (L1FR+, L2FR+). The data generally met the assumptions of residuals normality and of homoscedasticity for running parametric tests.

## Supplementary information

Supplementary Figure 1.

Supplementary Figure 2.

Supplementary Figure 3.

Supplementary Figure 4.

Supplementary Figure 5.

Supplementary Figure 6.

Supplementary Figure 7.

Supplementary Table S1.

Supplementary Table S2.

Supplementary Table S3.

Supplementary Table S4.

## Data Availability

All data generated or analysed during this study are included in this published article (and its Supplementary Information files).
