# Supplementary material for: Bryophytes can recognize their neighbours through volatile organic compounds
Source: Sci Rep. 2020 May 4;10:7405. doi: 10.1038/s41598-020-64108-y (PMC7198583; doi:10.1038/s41598-020-64108-y)
Supplement: Supplementary file 1 — Supplementary Figure 1. [file 41598_2020_64108_MOESM1_ESM.pdf]

Bryophytes can recognize their neighbours through volatile organic compounds

Eliška Vicherová, Robert Glinwood, TomášHájek, Petr Šmilauer and Velemir Ninkovic

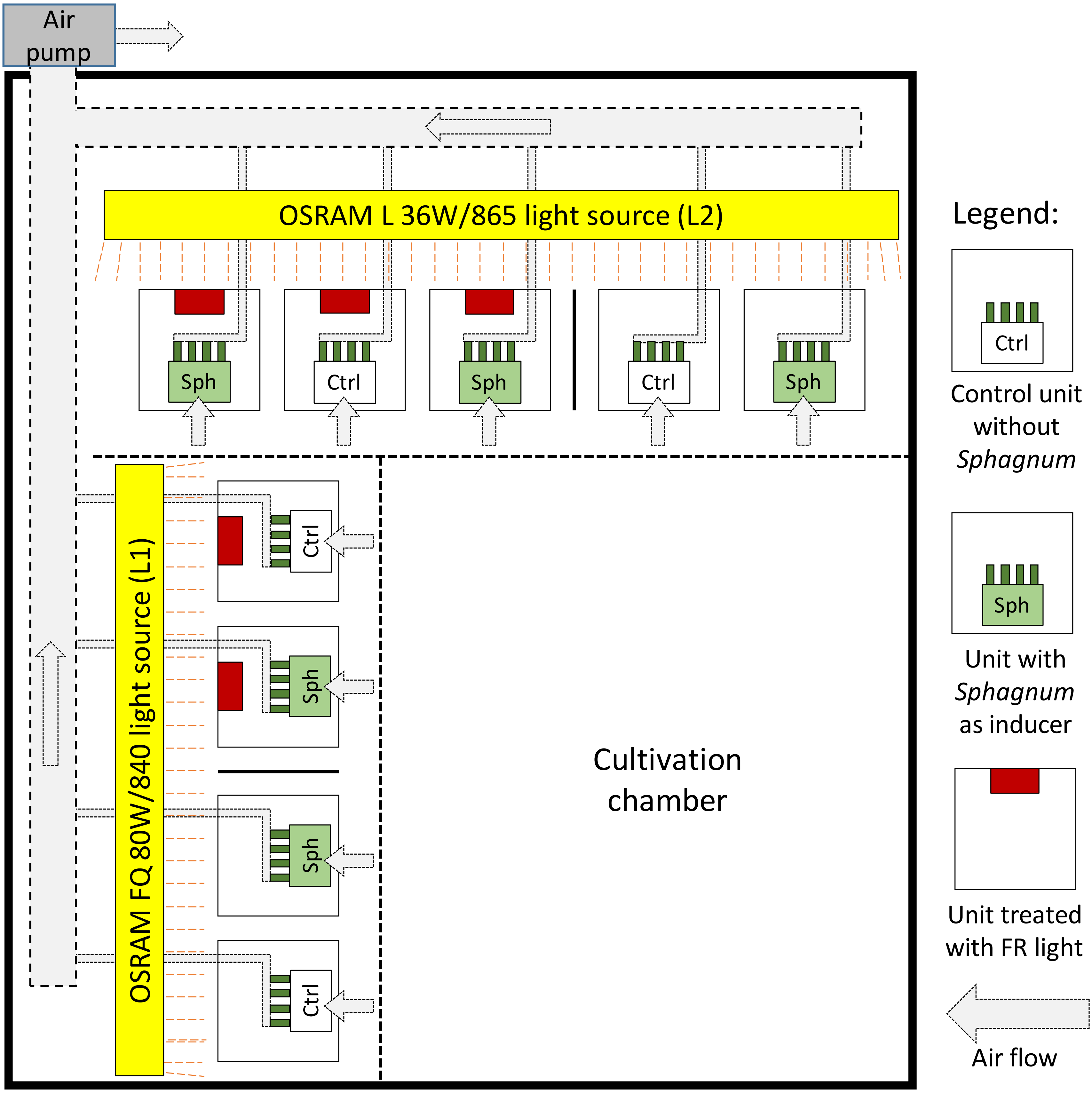

Supplemental Figure S1. The setup of cultivation units in the cultivation chamber during the experiment (the cultivation unit drafted in Fig.1). The units were illuminated by two sources of artificial daylight: Osram FQ 80W/840 (four units) and Osram L 36W/865 (five units). The FR light was added to five units; two were under Osram FQ 80W/840 (L1FR+ treatment), three under Osram L 36W/865 (L2FR+ treatment). Units with different light regimes were separated by non-transparent partitions. See Fig. S2 for spectral properties of the two light sources.
