# Supplementary material for: Bryophytes can recognize their neighbours through volatile organic compounds
Source: Sci Rep. 2020 May 4;10:7405. doi: 10.1038/s41598-020-64108-y (PMC7198583; doi:10.1038/s41598-020-64108-y)
Supplement: Supplementary file 2 — Supplementary Figure 2. [file 41598_2020_64108_MOESM2_ESM.pdf]

Bryophytes can recognize their neighbours through volatile organic compounds

Eliška Vicherová, Robert Glinwood, Tomáš Hájek, Petr Šmilauer and Velemir Ninkovic

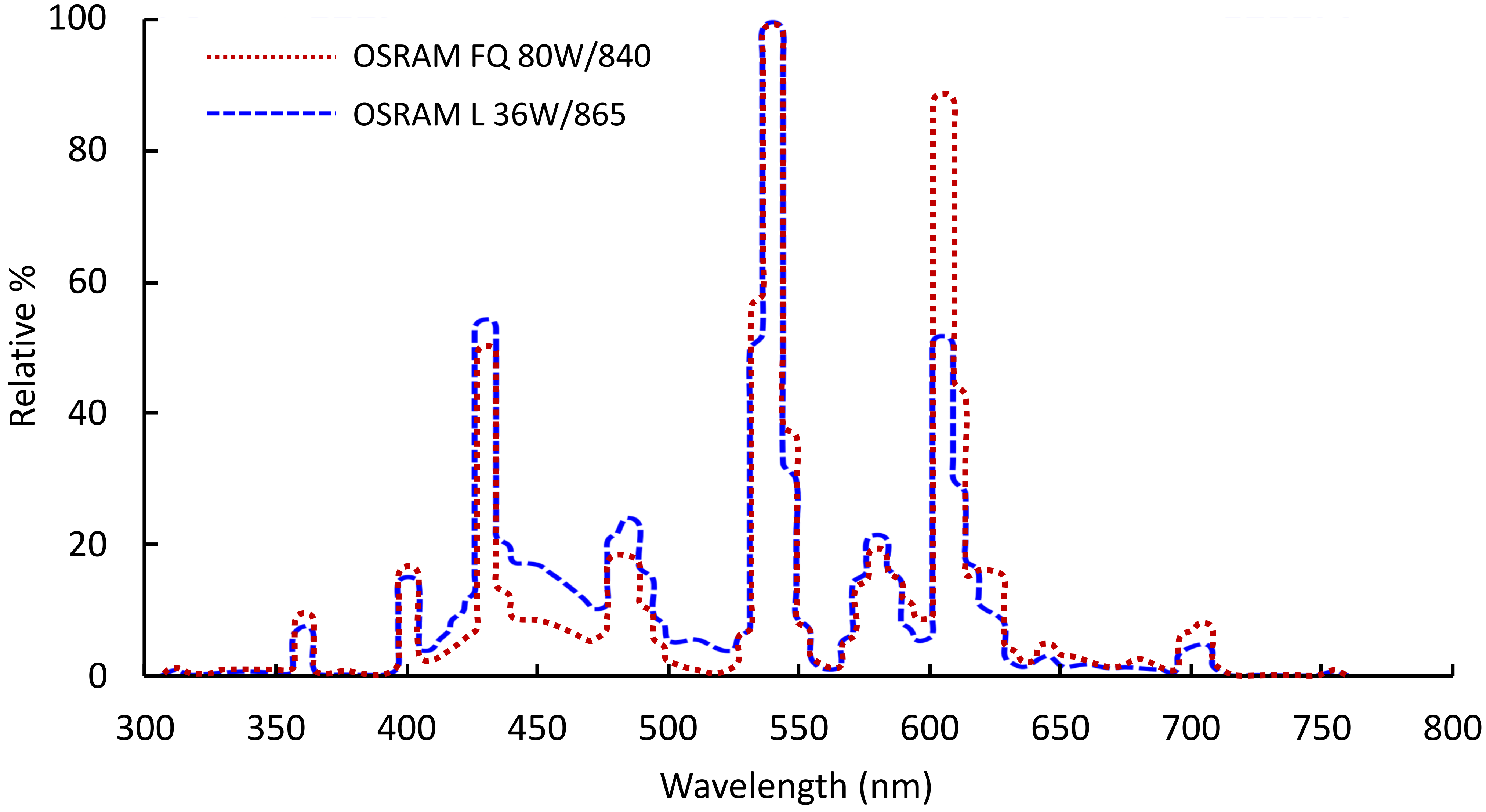

Supplemental Figure S2. Spectral power distribution diagram of two fluorescent Osram light sources used in the growth chamber. Redrawn from product datasheets (Osram GmbH, Germany).
