# Supplementary material for: Bryophytes can recognize their neighbours through volatile organic compounds
Source: Sci Rep. 2020 May 4;10:7405. doi: 10.1038/s41598-020-64108-y (PMC7198583; doi:10.1038/s41598-020-64108-y)
Supplement: Supplementary file 3 — Supplementary Figure 3. [file 41598_2020_64108_MOESM3_ESM.pdf]

Bryophytes can recognize their neighbours through volatile organic compounds

Eliška Vicherová, Robert Glinwood, Tomáš Hájek, Petr Šmilauer and Velemir Ninkovic

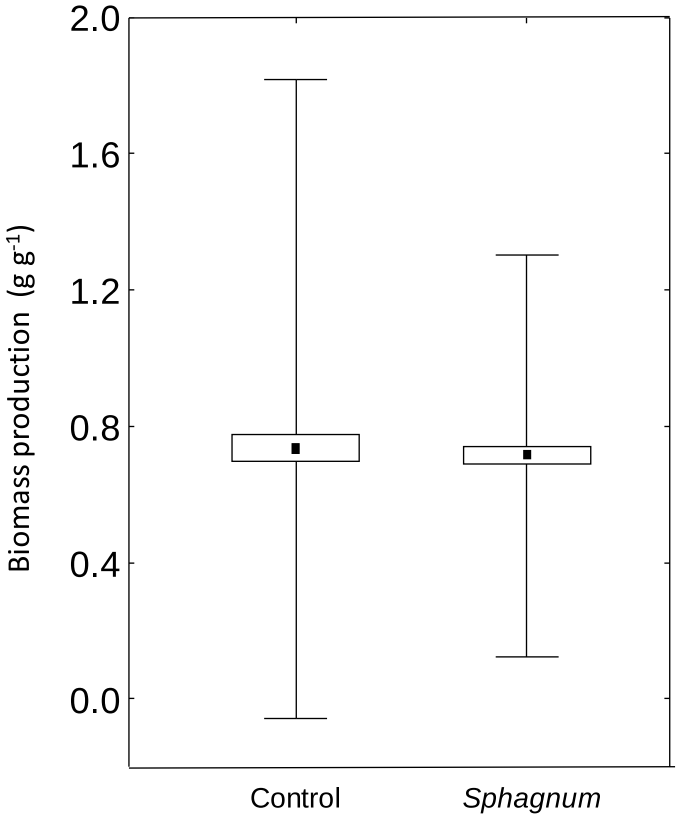

Supplemental Figure S3. The biomass production of *H. vernicosus* shoots grown in cultivation units (Fig. 1) for 30 days under different light treatments (light treatments were pooled together for the statistical analysis, see methods for details). The shoots were exposed to VOCs produced by surrounding *H. vernicosus* individuals and to VOCs from *S. flexuosum* chamber (*Sphagnum*) or chamber without *S. flexuosum* (*Control*). The box and whiskers depict ± s.e. and minimum/maximum values. The VOCs treatment has no effect on biomass production of *H. vernicosus* ($F_{1,7}$ = 6.8, p = 0.81).
