# Supplementary material for: Bryophytes can recognize their neighbours through volatile organic compounds
Source: Sci Rep. 2020 May 4;10:7405. doi: 10.1038/s41598-020-64108-y (PMC7198583; doi:10.1038/s41598-020-64108-y)
Supplement: Supplementary file 4 — Supplementary Figure 4. [file 41598_2020_64108_MOESM4_ESM.pdf]

Bryophytes can recognize their neighbours through volatile organic compounds

Eliška Vicherová, Robert Glinwood, Tomáš Hájek, Petr Šmilauer and Velemir Ninkovic

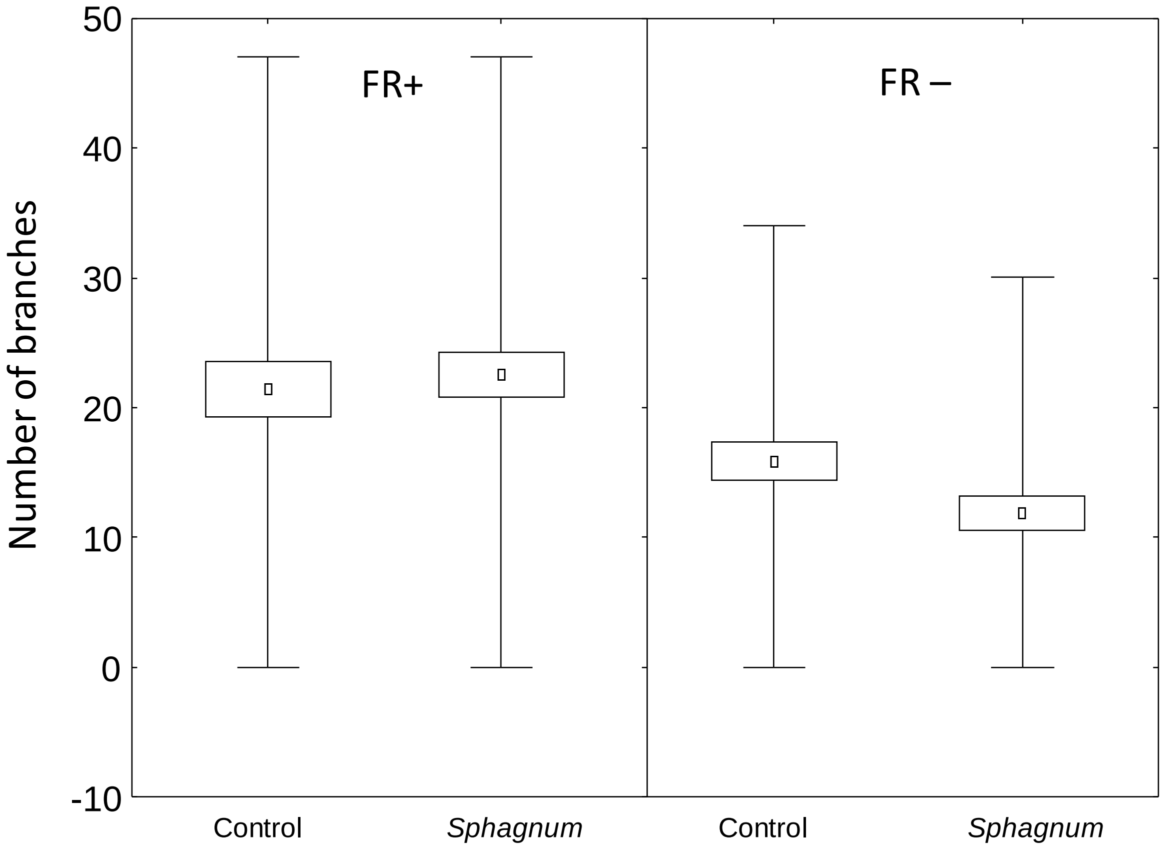

Supplemental Figure S4. The number of branches created by *H. vernicosus* shoots grown in cultivation units (Fig. 1) for 30 days under artificial light with and without FR light supplementation (FR+ and FR−). The shoots were exposed to VOCs produced by surrounding *H. vernicosus* individuals and to VOCs from *S. flexuosum* chamber (*Sphagnum*) or chamber without *S. flexuosum* (Control). The *S. flexuosum* VOCs had no effect on number of branches created by *H. vernicosus* under FR− ($F_{1,2}$ = 1.5, p = 0.34) or FR+ ($F_{1,3}$=0.06, p=0.82). The box and whiskers depict ± s.e. and minimum/maximum values.
