# Supplementary material for: Bryophytes can recognize their neighbours through volatile organic compounds
Source: Sci Rep. 2020 May 4;10:7405. doi: 10.1038/s41598-020-64108-y (PMC7198583; doi:10.1038/s41598-020-64108-y)
Supplement: Supplementary file 5 — Supplementary Figure 5. [file 41598_2020_64108_MOESM5_ESM.pdf]

Bryophytes can recognize their neighbours through volatile organic compounds

Eliška Vicherová, Robert Glinwood, Tomáš Hájek, Petr Šmilauer and Velemir Ninkovic

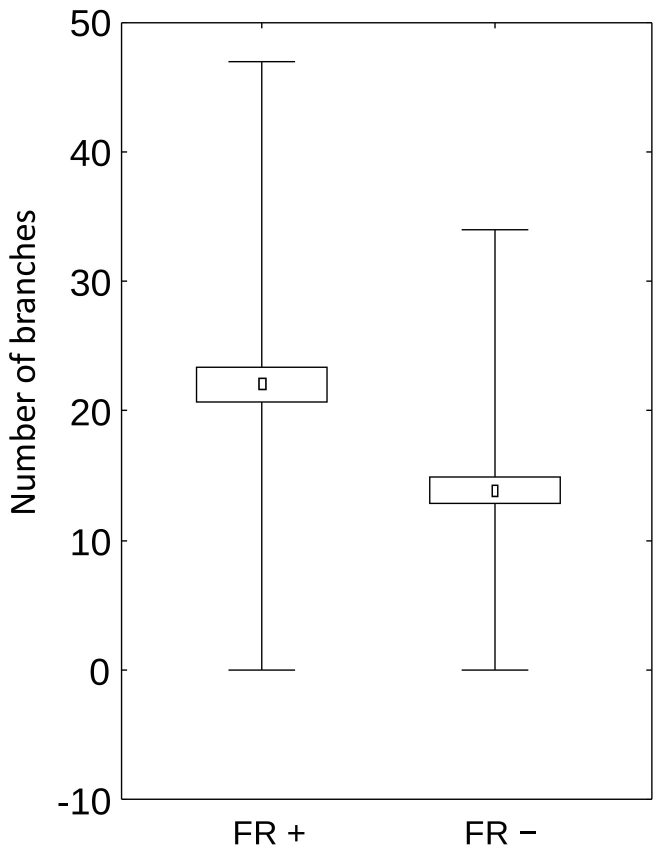

Supplemental Figure S5. The number of branches created by *H. vernicosus* shoots grown under artificial light with and without FR light supplementation (FR+ and FR−) in cultivation units (Fig. 1) for 30 days (FR+ includes L1 and L2 FR+, see methods for details). The shoots exposed and unexposed to *S. flexuosum* VOCs were pooled together for the statistical analysis. FR+ induced creation of more short branches ($F_{1,7}$=7.2, p=0.03). The box and whiskers depict ± s.e. and minimum/maximum values.
