# Supplementary material for: Bryophytes can recognize their neighbours through volatile organic compounds
Source: Sci Rep. 2020 May 4;10:7405. doi: 10.1038/s41598-020-64108-y (PMC7198583; doi:10.1038/s41598-020-64108-y)
Supplement: Supplementary file 6 — Supplementary Figure 6. [file 41598_2020_64108_MOESM6_ESM.pdf]

Bryophytes can recognize their neighbours through volatile organic compounds

Eliška Vicherová, Robert Glinwood, Tomáš Hájek, Petr Šmilauer and Velemir Ninkovic

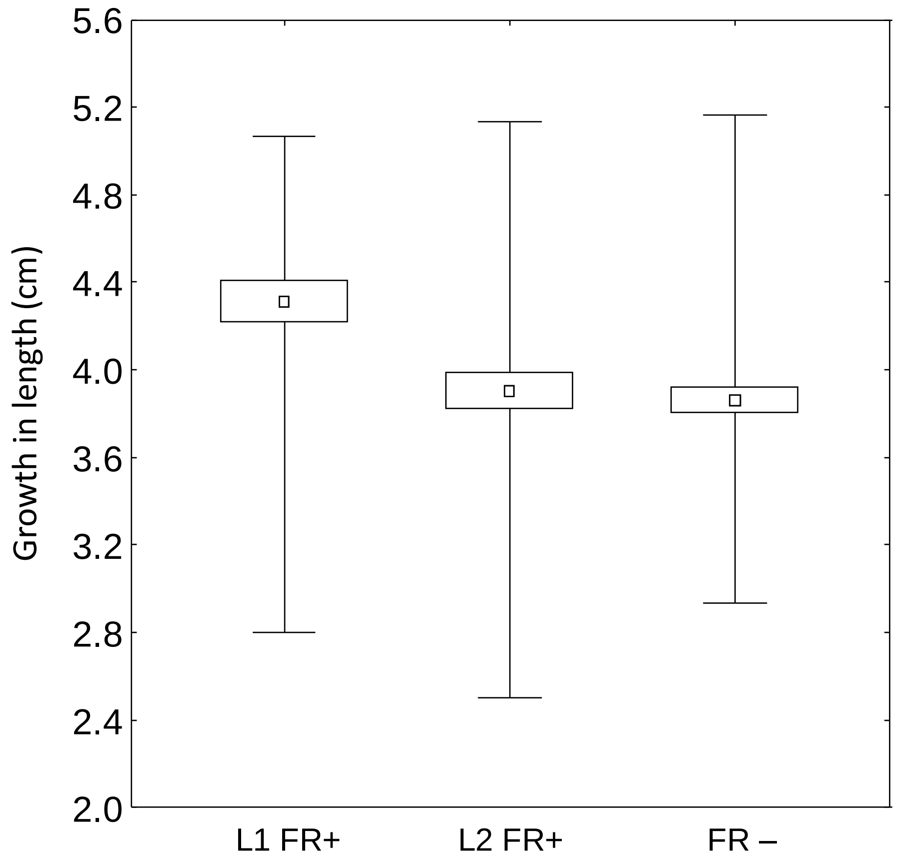

Supplemental Figure S6. The length increment of *H. vernicosus* shoots grown under artificial light without FR light addition (FR−) and added FR light (L1 FR+, L2 FR+) in cultivation units (Fig. 1) for 30 days (L2 FR+ had more blue light than L1 FR+, see methods and Fig. S2 for details). The shoots exposed and unexposed to *S. flexuosum* VOCs were pooled together for the statistical analysis. The *H. vernicosus* growth increment was not significantly affected by different light treatments ($F_{2,5}$=2.6, p=0.17, see also Fig. 3 for different data presentation). The box and whiskers depict ± s.e. and minimum/maximum values.
