# Supplementary material for: Bryophytes can recognize their neighbours through volatile organic compounds
Source: Sci Rep. 2020 May 4;10:7405. doi: 10.1038/s41598-020-64108-y (PMC7198583; doi:10.1038/s41598-020-64108-y)
Supplement: Supplementary file 7 — Supplementary Figure 7. [file 41598_2020_64108_MOESM7_ESM.pdf]

Bryophytes can recognize their neighbours through volatile organic compounds

Eliška Vicherová, Robert Glinwood, Tomáš Hájek, Petr Šmilauer and Velemir Ninkovic

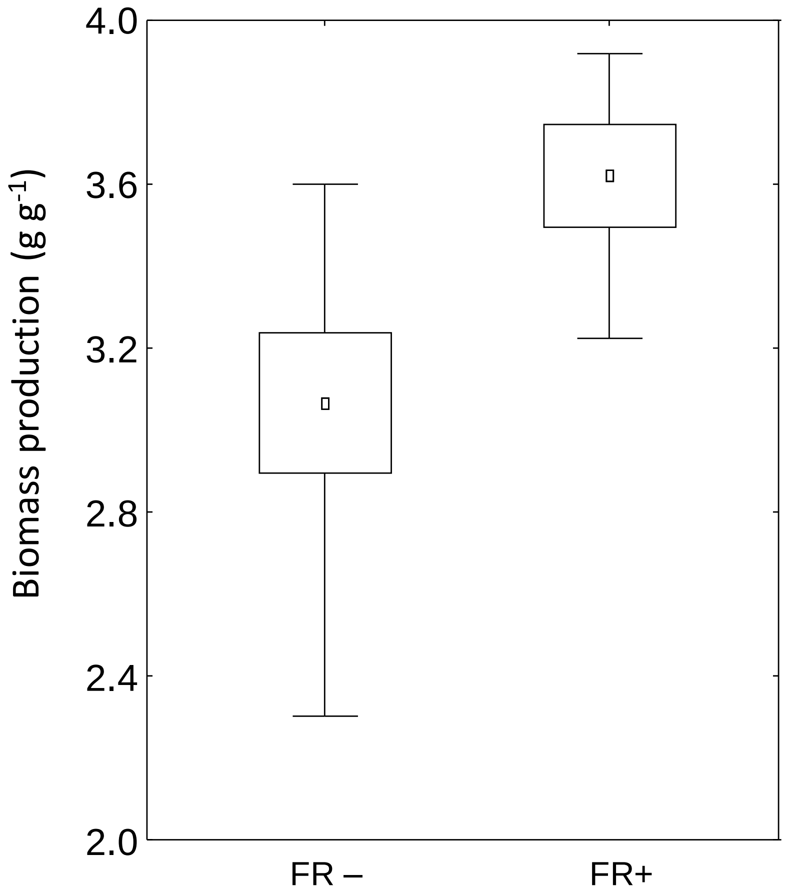

Supplemental Figure S7. The biomass production of *S.flexuosum* shoots grown in cultivation units (Fig. 1) for 30 days under artificial light without FR light addition (FR−) and added FR light (FR+; L1 and L2 FR+ treatments pooled together). The light treatment has no effect on biomass production of *S. flexuosum* ($F_{1,4}$=4.0, p=0.12; experimental design included in the test). The box and whiskers depict ± s.e. and minimum/maximum values.
