# Supplementary material for: Bryophytes can recognize their neighbours through volatile organic compounds
Source: Sci Rep. 2020 May 4;10:7405. doi: 10.1038/s41598-020-64108-y (PMC7198583; doi:10.1038/s41598-020-64108-y)
Supplement: Supplementary file 8 — Supplementary Table S1. [file 41598_2020_64108_MOESM8_ESM.pdf]

Bryophytes can recognize their neighbours through volatile organic compounds

Eliška Vicherová, Robert Glinwood, Tomáš Hájek, Petr Šmilauer and Velemir Ninkovic

Supplemental Table S1. Detailed description of source localities for the mosses *Sphagnum flexuosum* and *Hamatocaulis vernicosus* used in the experiments.

**Locality name**	**Mire type**	**Localization**	**GPS coordinates**	**Altitude (m a.s.l.)**	**pH**	**[Ca^{2+}] (mg L^{-1}) / κ (µS cm^{-1})**	**Sampled species**
Dlouhá louka	moderately rich fen	Plzeň region, Czech Republic	49°54'44"N 13°10'43"E	570	6.3	6.5/—	*Sphagnum flexuosum*
Hrádecká bahna	moderately rich fen	Plzeň region, Czech Republic	49°42'47"N 13°39'31"E	400	7.0	—/225	*Sphagnum flexuosum*
Řeka	rich fen	Vysočina region, Czech Republic	49°39'59"N 15°51'11"E	550	7.2	50/—	*Hamatocaulis vernicosus*
Bouskův mlýn	moderately rich fen meadow	České Budějovice region, Czech Republic	48°52'59"N 14°40'58"E	450	6.5	—/159	*Hamatocaulis vernicosus*
