# Supplementary material for: Bryophytes can recognize their neighbours through volatile organic compounds
Source: Sci Rep. 2020 May 4;10:7405. doi: 10.1038/s41598-020-64108-y (PMC7198583; doi:10.1038/s41598-020-64108-y)
Supplement: Supplementary file 9 — Supplementary Table S2. [file 41598_2020_64108_MOESM9_ESM.pdf]

Bryophytes can recognize their neighbours through volatile organic compounds

Eliška Vicherová, Robert Glinwood, Tomáš Hájek, Petr Šmilauer and Velemir Ninkovic

Supplemental Table S2. Volatile organic compounds (ng g^{-1}) produced by *Hamatocaulis vernicosus* carpets, exposed to *S. flexuosum* VOC (*Sphagnum*) or empty chamber (*control*), that were cultivated under standard light condition (FR–) of added FR light (FR+). The *H. vernicosus* shoots were exposed either to VOCs released from neighbouring *H. vernicosus* individuals or to *H. vernicosus* VOCs and VOCs coming from *S. flexuosum* carpets. For more detail see methods.

**Light**	**Control / *Sphagnum* exposure**	**Total**	β-cyclocitral	Methyl 2,6,6-trimethyl-1-cyclohexene-1-carboxylate	Unknown 1	Unknown 2	Unknown 3	α-copaene	Unknown 4	Unknown 5	(Z)-β-farnesene	(E)-β-farnesene	Unknown 6	Unknown 7
**FR+**	control	**121**	11.55	0.58	0.19	0.36	0.24	1.12	1.19	0.46	0.15	0.18	5.83	0.60
**FR+**	control	**130**	6.58	1.29	0.13	0.31	0.28	1.27	0.76	0.84	0.26	0.33	6.88	0.76
**FR+**	control	**392**	48.11	2.95	0.80	1.07	0.96	1.35	1.12	1.31	0.77	1.46	28.81	2.54
**FR+**	control	**257**	25.03	2.28	0.25	0.48	0.33	1.13	1.13	0.45	0.27	1.35	8.70	1.03
**mean**		**225**	**22.82**	**1.78**	**0.34**	**0.55**	**0.45**	**1.22**	**1.05**	**0.77**	**0.36**	**0.83**	**12.56**	**1.23**
**FR–**	control	**135**	6.35	0.09	0.06	0.11	0.34	1.21	0.34	0.72	0.28	0.12	0.22	0.48
**FR–**	control	**185**	9.18	0.08	0.25	0.38	0.28	1.41	1.27	0.74	0.35	0.49	8.16	0.81
**FR–**	control	**99**	5.56	0.22	0.10	0.20	0.10	0.61	0.28	0.57	0.17	0.19	3.57	0.42
**FR–**	control	**198**	7.85	0.30	0.18	0.54	0.43	1.96	0.85	0.49	0.54	0.80	9.58	1.03
**mean**		**168**	**7.23**	**0.17**	**0.15**	**0.31**	**0.29**	**1.30**	**0.68**	**0.63**	**0.33**	**0.40**	**5.38**	**0.69**
**FR+**	*Sphagnum*	**216**	18.56	2.00	0.20	0.43	0.41	1.65	1.10	0.59	0.32	0.72	9.51	0.72
**FR+**	*Sphagnum*	**497**	20.49	1.36	0.55	1.04	0.85	5.07	2.01	0.82	0.73	1.80	32.08	2.39
**FR+**	*Sphagnum*	**146**	21.69	3.15	0.10	0.26	0.60	0.48	0.29	0.40	0.16	0.72	2.91	0.66
**FR+**	*Sphagnum*	**170**	13.36	4.90	0.51	1.30	0.40	1.32	7.14	0.55	0.47	0.86	5.71	1.23
**FR+**	*Sphagnum*	**242**	22.66	1.77	0.28	0.54	0.51	2.17	1.24	0.36	0.35	1.50	11.19	0.94
**FR+**	*Sphagnum*	**380**	9.77	1.23	0.37	0.86	0.82	3.91	2.02	1.07	0.63	1.44	22.84	2.02
**mean**		**275**	**17.76**	**2.40**	**0.34**	**0.74**	**0.60**	**2.43**	**2.30**	**0.63**	**0.44**	**1.17**	**14.04**	**1.33**
**FR–**	*Sphagnum*	**150**	6.97	0.71	0.21	0.27	0.24	1.01	0.68	0.85	0.28	0.32	5.75	0.75
**FR–**	*Sphagnum*	**548**	18.82	1.55	0.63	1.17	1.00	4.87	3.12	1.54	0.92	2.07	29.64	2.12
**FR–**	*Sphagnum*	**150**	6.97	0.71	0.21	0.27	0.24	1.01	0.68	0.85	0.28	0.32	5.75	0.75
**FR–**	*Sphagnum*	**256**	12.95	0.50	0.32	0.60	0.44	2.46	1.14	0.59	0.39	1.34	14.13	1.03
**mean**		**276**	**11.43**	**0.87**	**0.34**	**0.58**	**0.48**	**2.34**	**1.40**	**0.96**	**0.47**	**1.01**	**13.82**	**1.16**

Table S2 (continued).

Light	Control / *Sphagnum* exposure	Unknown 8	Unknown 9	Unknown 10	Unknown 11	Unknown 12	Unknown 13	Unknown 14	Unknown 15	Unknown 16	Rimuene	Unknown 17	Unknown 18
**FR+**	control	0.95	5.04	16.61	5.50	1.48	2.87	1.19	11.11	0.43	11.60	41.48	0.70
**FR+**	control	1.39	9.39	11.62	13.06	1.83	3.84	0.74	8.22	0.81	49.13	9.33	0.87
**FR+**	control	6.31	37.57	67.55	27.26	6.93	14.53	5.66	27.98	6.84	97.72	1.82	0.93
**FR+**	control	2.82	18.57	31.78	12.36	5.03	9.85	3.99	25.11	0.85	69.11	33.56	1.80
**mean**		**2.87**	**17.64**	**31.89**	**14.55**	**3.82**	**7.77**	**2.89**	**18.10**	**2.23**	**56.89**	**21.55**	**1.08**
**FR–**	control	0.22	1.32	1.15	0.44	0.50	0.23	0.26	2.24	0.89	16.47	99.61	1.40
**FR–**	control	1.88	8.42	28.36	14.12	3.40	6.82	3.46	18.96	1.01	46.31	27.79	0.74
**FR–**	control	0.61	4.73	7.92	6.41	1.62	2.71	1.18	4.69	0.49	48.10	8.02	0.88
**FR–**	control	2.40	7.60	24.38	15.74	4.71	7.98	2.84	14.43	2.53	56.32	33.36	0.77
**mean**		**1.28**	**5.52**	**15.45**	**9.18**	**2.56**	**4.43**	**1.94**	**10.08**	**1.23**	**41.80**	**42.19**	**0.95**
**FR+**	*Sphagnum*	2.03	11.43	23.73	12.33	2.67	6.00	2.83	19.64	0.91	68.26	28.53	1.21
**FR+**	*Sphagnum*	7.29	47.06	79.95	73.30	7.04	14.70	6.39	55.79	3.43	51.16	81.22	0.86
**FR+**	*Sphagnum*	0.59	8.27	9.08	1.46	0.56	0.50	1.14	9.46	0.49	76.32	5.14	1.72
**FR+**	*Sphagnum*	1.42	11.99	4.59	14.26	1.68	4.37	0.81	5.14	4.35	79.92	1.04	2.58
**FR+**	*Sphagnum*	3.17	23.47	31.23	21.16	3.90	6.62	2.62	25.46	1.49	50.09	28.79	0.93
**FR+**	*Sphagnum*	5.22	31.47	57.51	39.46	7.88	15.08	4.16	47.97	3.15	18.92	101.04	1.30
**mean**		**3.29**	**22.28**	**34.35**	**26.99**	**3.95**	**7.88**	**2.99**	**27.25**	**2.30**	**57.45**	**40.96**	**1.43**
**FR–**	*Sphagnum*	1.59	7.76	14.42	8.79	2.24	4.27	1.40	12.39	0.84	56.20	20.99	1.15
**FR–**	*Sphagnum*	7.15	30.70	60.84	47.71	7.57	15.59	5.96	38.32	4.40	188.85	69.96	3.99
**FR–**	*Sphagnum*	1.59	7.76	14.42	8.79	2.24	4.27	1.40	12.39	0.84	56.20	20.99	1.15
**FR–**	*Sphagnum*	3.97	20.18	39.79	18.81	4.90	10.35	3.72	33.32	1.68	45.91	36.39	1.38
**mean**		**3.58**	**16.60**	**32.37**	**21.02**	**4.24**	**8.62**	**3.12**	**24.11**	**1.94**	**86.79**	**37.08**	**1.92**
