# Supplementary material for: Bryophytes can recognize their neighbours through volatile organic compounds
Source: Sci Rep. 2020 May 4;10:7405. doi: 10.1038/s41598-020-64108-y (PMC7198583; doi:10.1038/s41598-020-64108-y)
Supplement: Supplementary file 10 — Supplementary Table S3. [file 41598_2020_64108_MOESM10_ESM.pdf]

Bryophytes can recognize their neighbours through volatile organic compounds

Eliška Vicherová, Robert Glinwood, Tomáš Hájek, Petr Šmilauer and Velemir Ninkovic

Supplemental Table S3. Volatile organic compounds (ng g^{-1}) produced by *Sphagnum flexuosum* carpets, cultivated under standard light condition (FR–) of added FR light (FR+). The VOCs sampling was done either under standard light condition (SS) or the FR light was added to shoots exposed to FR light during cultivation (FRS).

**Sampling**	**Light**	**Total**	α-pinene	β-myrcene	(E)-ocimene	β-cyclocitral	Methyl 2,6,6-trimethyl-1-cyclohexene-1-carboxylate	(+)-cyclosativene	α-copaene	Unknown 4	(±)-geosmin	(+)-sativene	Unknown 19	Unknown 20	Unknown 21	Unknown 22	Unknown 23
SS	FR–	**121**	0.33	0.41	3.28	2.02	0.67	0.90	0.98	0.29	0.89	1.94	0.44	13.22	3.19	10.14	2.43
SS	FR–	**40**	0.63	0.32	0.60	1.06	0.20	0.23	0.48	0.32	0.20	0.61	0.13	3.68	0.63	2.30	0.57
SS	FR–	**13**	0.02	0.11	0.45	0.31	0.08	0.01	0.03	0.03	0.04	0.06	0.02	1.39	0.18	0.11	0.07
SS	FR–	**25**	0.04	0.20	0.69	0.55	0.14	0.07	0.09	0.08	0.08	0.17	0.04	2.31	0.70	0.15	0.51
**mean**		**50**	**0.25**	**0.26**	**1.26**	**0.99**	**0.27**	**0.30**	**0.39**	**0.18**	**0.30**	**0.69**	**0.16**	**5.15**	**1.18**	**3.17**	**0.90**
SS	FR+	**59**	0.03	0.29	1.27	5.94	2.03	0.02	0.18	0.16	0.10	0.04	0.13	4.49	1.29	0.19	4.19
SS	FR+	**70**	0.07	0.33	2.18	7.28	3.93	0.08	0.28	0.07	0.11	0.16	0.19	5.14	1.31	0.44	3.60
SS	FR+	**76**	0.06	0.21	0.93	4.79	2.77	0.10	0.30	0.08	0.23	0.28	0.20	7.05	1.66	0.41	2.89
SS	FR+	**85**	0.11	0.44	2.46	4.53	2.65	0.40	0.43	0.06	0.31	0.75	0.23	9.10	1.93	1.92	1.01
SS	FR+	**103**	0.05	0.28	1.30	5.64	3.00	0.23	0.32	0.12	0.27	0.59	0.36	8.34	1.72	2.31	2.42
SS	FR+	**83**	0.08	0.37	2.70	5.14	2.72	0.19	0.19	0.12	0.27	0.38	0.29	8.08	2.04	1.76	1.78
**mean**		**79**	**0.07**	**0.32**	**1.81**	**5.55**	**2.85**	**0.17**	**0.28**	**0.10**	**0.21**	**0.37**	**0.23**	**7.03**	**1.66**	**1.17**	**2.65**
FRS	FR–	**85**	0.58	1.42	14.94	1.14	0.51	1.46	1.02	0.38	0.81	3.00	0.71	10.26	3.46	6.30	5.53
FRS	FR–	**51**	0.03	0.10	0.50	0.52	0.14	0.78	1.70	0.46	0.33	1.73	0.30	3.01	1.96	9.29	3.18
FRS	FR–	**30**	0.08	0.57	2.41	0.47	0.22	0.34	0.06	0.08	0.21	0.78	0.15	2.71	0.57	1.26	0.72
FRS	FR–	**27**	0.03	0.21	1.06	1.11	0.37	0.11	0.10	0.12	0.08	0.28	0.10	2.10	0.56	0.69	2.39
**mean**		**48**	**0.18**	**0.57**	**4.72**	**0.81**	**0.31**	**0.67**	**0.72**	**0.26**	**0.35**	**1.45**	**0.32**	**4.52**	**1.64**	**4.38**	**2.96**
FRS	FR+	**60**	0.09	0.58	3.19	3.35	2.21	0.09	0.30	0.08	0.10	0.27	0.31	4.18	1.14	0.68	8.20
FRS	FR+	**95**	0.16	0.87	2.75	8.87	5.76	0.23	0.60	0.13	0.14	0.17	0.46	5.73	1.29	0.47	2.64
FRS	FR+	**68**	0.10	0.55	2.68	3.49	2.70	0.18	0.26	0.08	0.15	0.41	0.25	5.32	1.00	1.12	6.02
FRS	FR+	**100**	0.08	0.66	3.55	4.41	1.97	0.10	0.46	0.16	0.10	0.19	0.15	3.89	1.48	0.50	32.80
FRS	FR+	**86**	0.09	0.65	3.50	4.32	2.46	0.12	0.38	0.08	0.13	0.15	0.27	3.88	1.62	0.44	26.86
FRS	FR+	**47**	0.02	0.17	1.01	2.71	1.05	0.04	0.22	0.14	0.16	0.09	0.15	1.63	0.63	0.21	15.88
**mean**		**76**	**0.09**	**0.58**	**2.78**	**4.52**	**2.69**	**0.13**	**0.37**	**0.11**	**0.13**	**0.21**	**0.26**	**4.11**	**1.19**	**0.57**	**15.40**

Supplemental Table S3 (continued).

Sampling	Light	Unknown 24	(-)-calamene ne	Unknown 25	Unknown 26	Unkown 27	Unknown 28	Unknown 29	Unkown 30	Unknown 31	Unknown 32	Unknown 33	Unknown 34	Manoyl oxide	Unknown 35
SS	FR–	2.89	0.92	0.96	11.33	0.76	0.12	0.35	0.66	5.25	5.18	2.10	39.64	5.17	4.93
SS	FR–	1.24	0.30	0.58	3.55	0.24	0.07	0.19	0.24	4.37	2.01	0.15	11.08	1.95	2.29
SS	FR–	0.36	0.04	0.06	1.53	0.09	0.02	0.07	0.11	0.99	0.68	0.08	3.82	0.71	1.05
SS	FR–	0.29	0.14	0.07	2.82	0.15	0.10	0.16	0.33	1.29	1.24	0.43	9.09	1.49	2.03
**mean**		**1.19**	**0.35**	**0.42**	**4.81**	**0.31**	**0.08**	**0.19**	**0.33**	**2.97**	**2.28**	**0.69**	**15.91**	**2.33**	**2.58**
SS	FR+	1.03	0.09	0.22	4.28	0.40	0.43	1.04	1.80	5.46	1.73	0.74	16.01	2.28	3.50
SS	FR+	1.32	0.10	0.21	4.29	0.30	0.73	1.68	2.47	6.18	2.18	1.57	18.02	2.50	2.98
SS	FR+	1.68	0.09	0.22	5.22	0.39	0.54	1.32	1.94	6.20	2.64	2.38	25.11	2.78	3.51
SS	FR+	2.00	0.44	0.31	6.36	0.29	0.44	0.90	1.34	6.65	2.94	0.81	29.62	3.47	3.42
SS	FR+	1.63	0.26	0.29	6.70	0.17	0.42	0.87	1.53	15.00	3.19	2.25	33.87	3.44	5.98
SS	FR+	1.56	0.27	0.37	6.06	0.02	0.26	0.61	0.99	9.51	2.64	0.27	26.76	3.18	3.96
**mean**		**1.54**	**0.21**	**0.27**	**5.48**	**0.26**	**0.47**	**1.07**	**1.68**	**8.17**	**2.55**	**1.34**	**24.90**	**2.94**	**3.89**
FRS	FR–	3.04	0.70	0.90	6.57	0.15	0.27	0.21	0.91	2.35	4.68	0.74	4.15	5.04	3.27
FRS	FR–	0.95	1.93	0.46	2.33	0.10	0.37	0.09	0.48	2.66	1.42	0.58	12.53	1.47	1.98
FRS	FR–	0.75	0.14	0.10	2.23	0.16	0.03	0.12	0.44	1.04	1.58	0.10	10.12	1.67	1.09
FRS	FR–	0.61	0.11	0.14	1.70	0.04	0.04	0.27	0.26	0.85	1.15	0.65	9.57	1.23	1.40
**mean**		**1.34**	**0.72**	**0.40**	**3.21**	**0.11**	**0.18**	**0.17**	**0.52**	**1.72**	**2.21**	**0.51**	**9.09**	**2.35**	**1.94**
FRS	FR+	0.94	0.13	0.25	3.10	0.41	0.75	1.41	3.07	4.94	2.21	4.04	10.16	1.91	2.14
FRS	FR+	1.54	0.16	0.36	6.15	0.79	3.20	5.90	11.64	7.70	3.31	1.59	15.18	3.60	3.37
FRS	FR+	1.19	0.23	0.27	3.40	0.83	0.89	1.57	2.87	6.43	2.21	6.38	12.82	1.98	2.82
FRS	FR+	1.01	0.11	0.39	2.25	1.21	2.11	2.69	6.00	4.08	2.12	11.63	11.89	1.89	2.05
FRS	FR+	1.02	0.08	0.49	2.12	1.14	1.47	3.19	5.46	3.72	2.30	5.26	11.38	1.57	2.20
FRS	FR+	0.50	0.23	0.06	0.95	1.05	1.08	1.63	3.67	2.01	0.88	3.81	5.03	0.78	1.10
**mean**		**1.03**	**0.16**	**0.30**	**2.99**	**0.91**	**1.58**	**2.73**	**5.45**	**4.81**	**2.17**	**5.45**	**11.08**	**1.95**	**2.28**
