# Supplementary material for: Bryophytes can recognize their neighbours through volatile organic compounds
Source: Sci Rep. 2020 May 4;10:7405. doi: 10.1038/s41598-020-64108-y (PMC7198583; doi:10.1038/s41598-020-64108-y)
Supplement: Supplementary file 11 — Supplementary Table S4. [file 41598_2020_64108_MOESM11_ESM.pdf]

Bryophytes can recognize their neighbours through volatile organic compounds

Eliška Vicherová, Robert Glinwood, Tomáš Hájek, Petr Šmilauer and Velemir Ninkovic

Supplemental Table S4. Volatile organic compounds collected from *Sphagnum flexuosum* and *Hamatocaulis vernicosus* . Tentative compound identification, retention time and retention index (Kovats index) on HP-1 column, mass spectral data (m/z and relative abundance).

Tentative compound identification	Retention time (min)	KI (HP-1)	m/z fragments	Suggested chemical class of unknown	Occurs in species
β-cyclocitral	11.84	1201	137 (100), 152 (86), 109 (74), 123 (74), 81 (58), 67 (56), 41 (39), 91 (34), 79 (31), 77 (27)		*Hamatocaulis vernicosus, Sphagnum flexuosum*
Methyl 2,6,6-trimethyl-1-cyclohexene-1-carboxylate	12.10	1220	135 (100), 123 (52), 107 (51), 167 (36), 91 (23), 79 (22), 41 (18), 81 (16), 151 (16), 77 (15)		*Hamatocaulis vernicosus, Sphagnum flexuosum*
Unknown 1	13.88	1354	105 (100), 119 (82), 161 (75), 91 (53), 93 (39), 121 (32), 120 (28), 81 (25), 41 (25), 133 (18), 204	sesquiterpene	*Hamatocaulis vernicosus*
Unknown 2	13.94	1359	119 (100), 41 (59), 55 (48), 105 (44), 133 (39), 91 (35), 56 (30), 93 (25), 120 (23), 107 (23), 204	sesquiterpene	*Hamatocaulis vernicosus*
Unknown 3	14.18	1377	105 (100), 119 (92), 93 (74), 120 (67), 161 (54), 91 (53), 49 (45), 41 (39), 92 (35), 121 (27), 204	sesquiterpene	*Hamatocaulis vernicosus*
α-copaene	14.24	1382	119 (100, 105 (98), 161 (79), 93 (48), 91 (45), 41 (30), 92 (27), 81 (24), 120 (22), 77 (21), 204		*Hamatocaulis vernicosus, Sphagnum flexuosum*
Unknown 4	14.37	1392	161 (100), 41 (54), 105 (46), 55 (41), 57 (37), 91 (34), 81 (27), 79 (24), 67 (23), 120 (23), 204	sesquiterpene	*Hamatocaulis vernicosus, Sphagnum flexuosum*
Unknown 5	14.76	1424	105 (100), 161 (76), 91 (63), 119 (59), 107 (51), 189 (45), 93 (42), 204 (41), 79 (39), 41 (38)	sesquiterpene	*Hamatocaulis vernicosus*
(Z)-β-farnesene	14.96	1440	69 (100), 41 (67), 93 (54), 91 (38), 79 (34), 55 (31), 77 (30), 92 (29), 161 (28), 120 (26), 204		*Hamatocaulis vernicosus*
(E)-β-farnesene	15.07	1449	69 (100), 41 (91), 93 (58), 91 (35), 105 (33), 55 (29), 79 (29), 133 (28), 67 (28), 161 (26), 204		*Hamatocaulis vernicosus*
Unknown 6	15.21	1461	119 (100), 121 (87), 93 (70), 79 (56), 91 (45), 189 (42), 105 (41), 41 (40), 81 (37), 77 (33), 204	sesquiterpene	*Hamatocaulis vernicosus*
Unknown 7	15.28	1467	43 (100), 119 (94), 177 (88), 149 (77), 93 (69), 91 (68), 121 (64), 105 (57), 77 (42), 79 (40), 204	sesquiterpene	*Hamatocaulis vernicosus*
Unknown 8	15.37	1475	119 (100), 121 (68), 105 (62), 93 (59), 91 (58), 79 (49), 41 (48), 81 (42), 107 (38), 189 (37), 204	sesquiterpene	*Hamatocaulis vernicosus*
Unknown 9	15.60	1493	161 (100), 105 (56), 119 (44), 91 (43), 41 (31), 43 (24), 81 (21), 55 (19), 93 (18), 79 (16), 204	sesquiterpene	*Hamatocaulis vernicosus*
Unknown 10	15.83	1512	161 (100), 43 (64), 105 (62), 119 (50), 91 (46), 41 (40), 81 (37), 93 (37), 55 (31), 79 (31), 204	sesquiterpene	*Hamatocaulis vernicosus*
Unknown 11	15.95	1523	105(100), 43 (99), 220 (76), 91 (76), 106 (70), 81 (69), 41 (54), 93 (50), 147 (47), 137 (46)		*Hamatocaulis vernicosus*
Unknown 12	16.07	1534	108 (100), 126 (99), 43 (95), 81 (60), 82 (47), 41 (47), 55 (46), 109 (39), 67 (37), 83 (35)		*Hamatocaulis vernicosus*
Unknown 13	16.35	1558	55 (100), 41 (96), 137 (82), 109 (80), 81 (77), 43 (72), 207 (67), 95 (61), 149 (51), 107 (49)		*Hamatocaulis vernicosus*
Unknown 14	16.64	1583	161 (100), 105 (59), 207 (59), 43 (54), 91 (38), 119 (37), 41 (30), 81 (28), 93 (19), 55 (18)		*Hamatocaulis vernicosus*
Unknown 15	17.02	1617	161 (100), 59 (72), 81 (59), 93 (49), 204 (42), 79 (42), 119 (41), 91 (39), 105 (39), 41 (34)		*Hamatocaulis vernicosus*
Unknown 16	19.31	1835	161 (100), 91 (22), 105 (20), 41 (20), 119 (15), 133 (14), 107 (14), 93 (13), 162 (13), 147 (12)		*Hamatocaulis vernicosus*
Rimuene	20.30	1937	257 (100), 80 (58), 91 (42), 93 (42), 81 (42), 121 (40), 55 (39), 41 (38), 79 (39), 105 (37), 272	diterpene	*Hamatocaulis vernicosus*
Unknown 17	20.64	1972	161 (100), 41 (45), 82 (40), 55 (34), 105 (32), 69 (28), 93 (28), 91 (27), 121 (24), 43 (23)		*Hamatocaulis vernicosus*
Unknown 18	21.33	2047	91 (100), 41 (78), 105 (77), 55 (61), 69 (60), 79 (59), 81 (59), 133 (58), 95 (54), 123 (51)		*Hamatocaulis vernicosus*
α-pinene	7.89	941	93 (100), 91 (43), 92 (37), 77 (32), 79 (25), 41 (18), 105 (15), 121 (13), 94 (11), 80 (11), 136		*Sphagnum flexuosum*
β-myrcene	8.66	988	93 (100), 41 (93), 69 (71), 91 (23), 79 (17), 77 (15), 53 (14), 67 (13), 92 (11), 94 (10), 136		*Sphagnum flexuosum*
(E)-ocimene	9.33	1032	93 (100), 91 (45), 92 (40), 79 (36), 77 (31), 41 (24), 106 (16), 80 (16), 55 (15), 121 (11), 136		*Sphagnum flexuosum*
(+)-cyclosativene	14.15	1375	105 (100), 91 (72), 119 (71), 161 (71), 94 (66), 120 (50), 107 (49), 93 (47), 41 (43), 133 (33), 204		*Sphagnum flexuosum*
(±)-geosmin	14.42	1395	112 (100), 55 (23), 41 (23), 111 (21), 43 (18), 108 (18), 125 (14), 93 (13), 126 (12), 97 (12), 182		*Sphagnum flexuosum*
(+)-sativene	14.50	1401	108 (100), 91 (87), 161 (78), 105 (76), 93 (58), 119 (57), 147 (48), 79 (44), 133 (44), 41 (43), 204		*Sphagnum flexuosum*
Unknown 19	14.75	1423	161 (100), 119 (53), 189 (50), 105 (49), 204 (35), 91 (35), 162 (33), 147 (28), 133 (27), 41 (25)	sesquiterpene	*Sphagnum flexuosum*
Unknown 20	14.89	1434	147 (100), 105 (94), 91 (53), 93 (43), 119 (41), 107 (33), 41 (30), 190 (30), 79 (30), 175 (29)		*Sphagnum flexuosum*
Unknown 21	15.19	1460	175 (100), 105 (33), 91 (31), 119 (31), 93 (23), 121 (23), 41 (23), 190 (21), 133 (20), 95 (19)		*Sphagnum flexuosum*
Unknown 22	15.41	1477	43 (100), 137 (92), 109 (50), 93 (49), 161 (47), 41 (43), 81 (43), 121 (41), 105 (39), 95 (38), 222		*Sphagnum flexuosum*
Unknown 23	15.65	1496	121 (100), 93 (84), 105 (81), 107 (55), 91 (54), 41 (46), 79 (43), 119 (38), 161 (36), 94 (34), 204	sesquiterpene	*Sphagnum flexuosum*
Unknown 24	15.74	1504	173 (100), 188 (16), 174 (14), 128 (12), 143 (9), 129 (9), 158 (9), 115 (9), 145 (8), 141 (8)		*Sphagnum flexuosum*
(-)-calamenene	15.85	1514	159 (100), 160 (13), 128 (13), 129 (13), 131 (10), 144 (9), 202 (8), 115 (8), 143 (6), 105 (6)		*Sphagnum flexuosum*
Unknown 25	15.90	1519	161 (100), 119 (76), 105 (70), 134 (68), 91 (50), 204 (40), 41 (33), 81 (30), 133 (23), 162 (23)	sesquiterpene	*Sphagnum flexuosum*
Unknown 26	16.44	1566	43 (100), 121 (86), 109 (81), 175 (56), 105 (55), 93 (54), 190 (49), 107 (44), 91 (41), 108 (39)		*Sphagnum flexuosum*
Unkown 27	16.51	1572	43 (100), 91 (94), 119 (82), 159 (80), 205 (78), 131 (64), 41 (54), 145 (50), 105 (46), 117 (46)	sesquiterpenoid	*Sphagnum flexuosum*
unknown 28	16.57	1577	43 (100), 91 (63), 41 (57), 79 (48), 105 (45), 81 (40), 159 (40), 107 (39), 96 (38), 69 (36), 220	sesquiterpenoid	*Sphagnum flexuosum*
Unknown 29	16.61	1581	43 (100), 41 (65) 105 (65), 107 (61), 93 (55), 91 (55), 69 (48), 109 (47), 55 (45), 81 (44), 220, 222	sesquiterpenoid	*Sphagnum flexuosum*
Unkown 30	16.70	1588	43 (100), 107 (92), 41 (75), 109 (73), 93 (70), 105 (64), 81 (63), 69 (62), 91 (59), 161 (54), 222	sesquiterpenoid	*Sphagnum flexuosum*
Unknown 31	16.76	1593	109 (100), 43 (86), 136 (76), 121 (64), 93 (51), 147 (47), 105 (45), 175 (42), 91 (41), 41 (35), 208		*Sphagnum flexuosum*
Unknown 32	17.67	1676	95 (100), 107 (40), 41 (25), 123 (21), 55 (21), 121 (21), 91 (20), 93 (18), 81 (16), 79 (15), 220		*Sphagnum flexuosum*
Unknown 33	18.26	1732	41 (100), 91 (85), 105 (71), 67 (71), 55 (67), 79 (64), 93 (58), 81 (57), 95 (55), 43 (52)		*Sphagnum flexuosum*
Unknown 34	20.83	1992	95 (100), 107 (89), 191 (43), 121 (38), 81 (36), 55 (31), 79 (30), 41 (29), 93 (29), 91 (27)		*Sphagnum flexuosum*
Manoyl oxide	21.06	2017	43 (100), 55 (86), 81 (86), 95 (69), 67 (65), 257 (65), 41 (64), 69 (60), 275 (54), 137 (51), 290		*Sphagnum flexuosum*
Unknown 35	21.99	2121	95 (100), 107 (34), 55 (17), 121 (15), 191 (15), 41 (13), 93 (12), 91 (12), 81 (10), 79 (10)		*Sphagnum flexuosum*

Tentative identification, mass spectrum and KI match with autentic standard; Speculative identification, strong match in commercial library, no standard available; Unknown compound, no satisfactory match in commercial library
